# Supplementary material for: The non-canonical SMC protein SmcHD1 antagonises TAD formation and compartmentalisation on the inactive X chromosome
Source: Nat Commun. 2019 Jan 3;10:30. doi: 10.1038/s41467-018-07907-2 (PMC6318279; doi:10.1038/s41467-018-07907-2)
Supplement: Supplementary file 1 — Supplementary Information [file 41467_2018_7907_MOESM1_ESM.pdf]

Supplementary Information

The non-canonical SMC protein SmcHD1 antagonises TAD formation and compartmentalisation on the inactive X chromosome

Gdula, Nesterova et al.

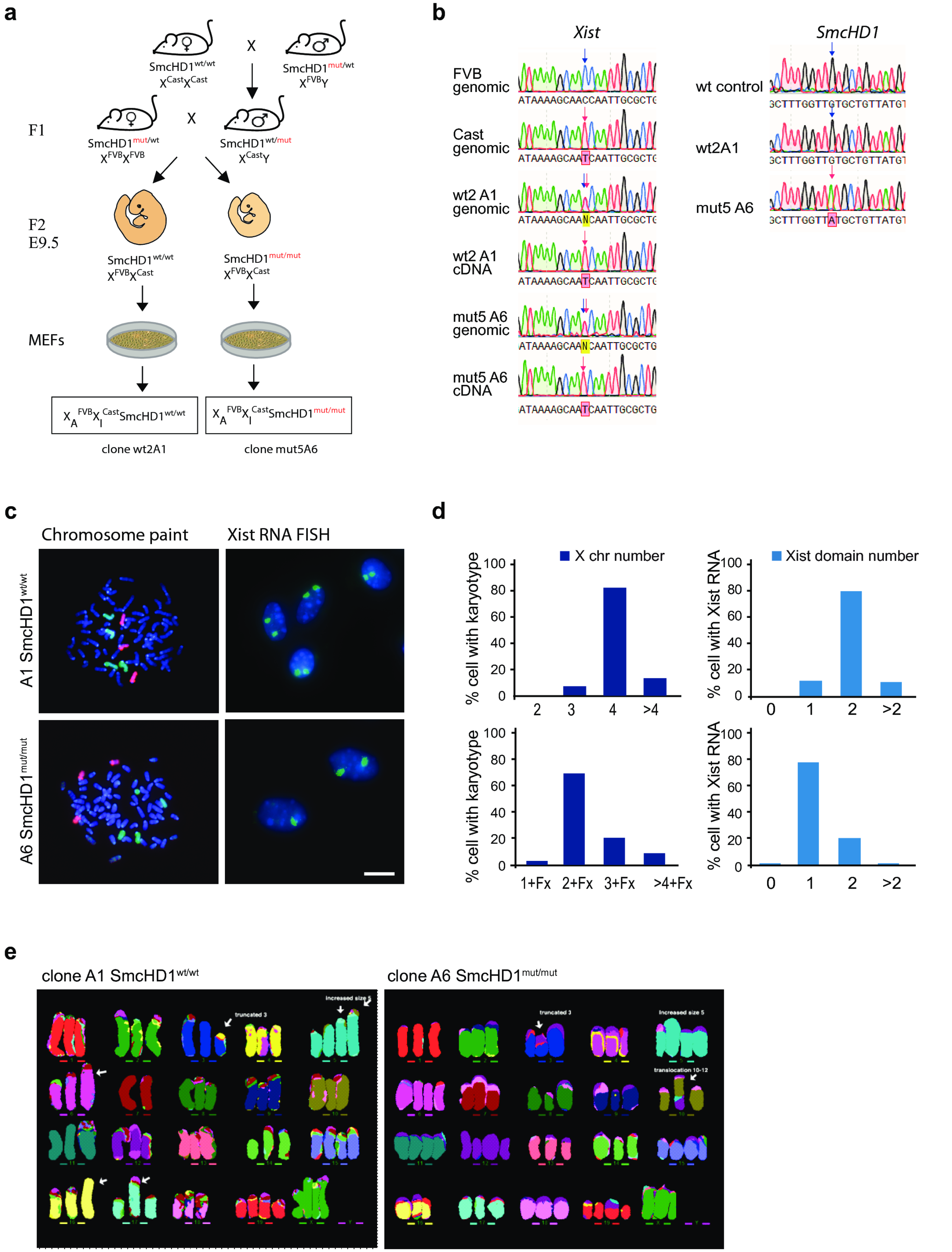

Supplementary Fig. 1 A MEF cell model for allele specific analysis of Xi/Xa. **a** Schematic

indicating genotypes and direction of crosses used to generate F2 embryos for MEF lines. Established MEF lines were genotyped and subcloned to isolate clonal lines carrying either castaneus (cast) or domesticus (FVB) Xi. **b** Left; sequencing traces of FVB, castaneus and F2 progeny Xist PCR fragments encompassing SNP region. Genomic and cDNA traces are shown to identify the origin of Xi for the cell lines studied. SNP is indicated in blue (C, FVB) or red (T, Cast) arrow. Right; sequencing traces of wt and mutant SmcHD1 PCR fragments. wt (G, blue arrow) and SmcHD1 mutant (mut) (A, red arrow) SNPs are indicated. **c** Karyotype analysis of the wt (clone A1, top) and mut (clone A6, bottom) cell lines. Total number of X chromosomes detected by Chr X paint is shown in green and Chr 8 in red. Xist RNA FISH shows the number of inactive X chromosomes. Bar, 10 μm. **d** Quantification of the karyotype and Xist RNA FISH analyses. At least 32 metaphase spreads and over 130 cells were scored for karyotyping and RNA FISH, respectively. **e** Examples of multiplex in situ hybridisation (M-FISH) karyotypes of the wt (left) and mut (right) cell lines.

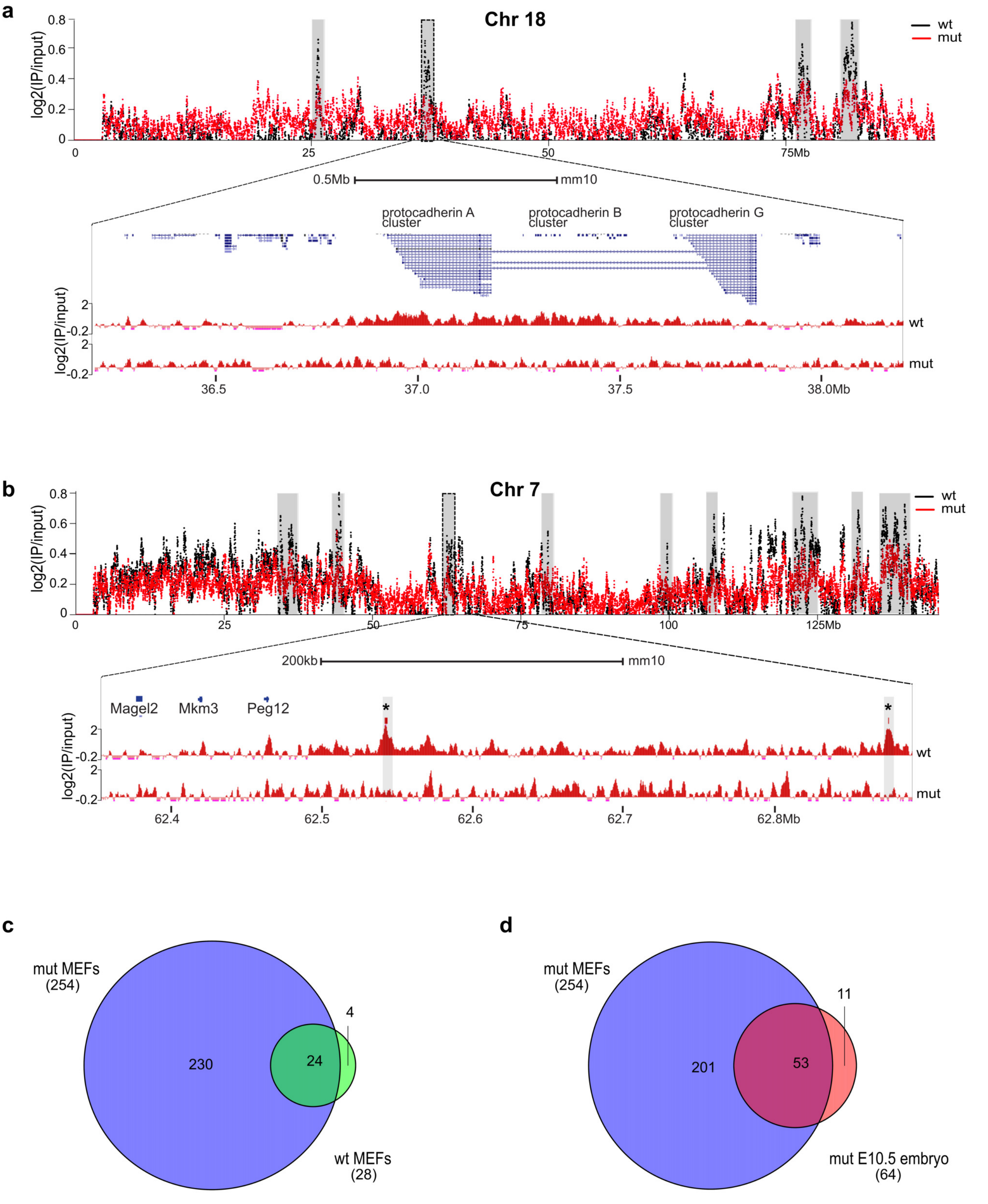

Supplementary Fig. 2 Analysis of SmcHD1 occupancy and transcription on Xi **a** Chromosome-wide profiles of SmcHD1 occupancy on Chr18 as allele-specific ChIP-seq log2 ratios of IP to input per 500 kb binned into 10 kb intervals. Profiles from wt (black) and SmcHD1 mutant (mut) MEFs (red) are presented. Regions enriched for SmcHD1 are shaded with magnification shown for the protocadhedrin cluster. **b** Chromosome-wide profiles of SmcHD1 on Chr 7 presented as in (a). Magnified region covers the imprinted gene cluster Magel2, Mkrn3, and Peg12. Specific peaks indicated with, small red boxes above the tracks, asterisks

were also reported in an independent study[37]. **c** Genes expressed from Xi in mut and wt MEFs. Total number for each cell type is shown in brackets. **d** Comparison of genes expressed from Xi in mut MEFs in this study, and in embryonic E10.5 cells with the same mutation, as published previously[34]. Total number for each study is shown in brackets.

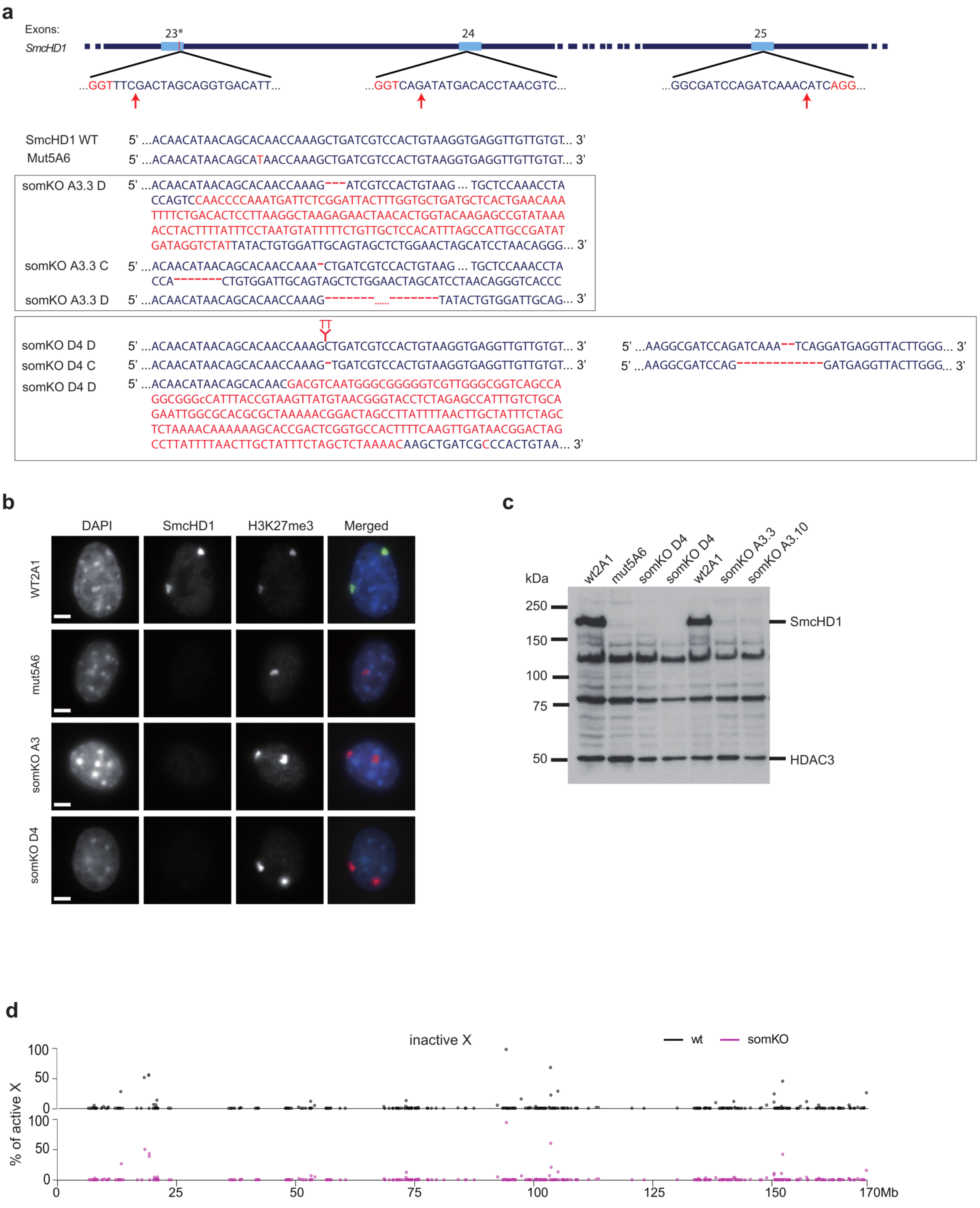

Supplementary Fig. 3 Generation and characterisation of SmcHD1 somatic knockout MEF lines. **a** Strategy for acute CRISPR-Cas9-mediated mutagenesis of *SmcHD1* gene. Relative position of the MommeD1 point mutation is indicated by the vertical red bar at the distal part of the exon 23. The positions of guide RNAs relative to *SmcHD1* exons are shown, with PAM sequences (red font) and predicted cleavage sites (red arrows) indicated. Two sgRNAs for exons 23 and 24 were used to generate a mutant clone A3.3 and two sgRNAs for exons 23

and 25 were used to generate clone D4. An alignment of wt with mutated sequences of *domesticus* (D) and *castaneus* (C) alleles of the clones A3.3 and D4 is shown below. As both clones are triploids, three independently mutated alleles are shown for each clone. Deletions are shown by red dashes and insertions/mutations are shown in red font. **b** Immunofluorescence analysis of SmcHD1 and H3K27me3 in wt, SmcHD1 *null* (mut) and SmcHD1 somatic knockout (somKO) MEF lines. Images show representative examples of cells with H3K27me3 Xi domains in all cell lines and loss of SmcHD1 Xi domains in mut and somKO cells. Scale bar is 5 µm. **c** Western blot analysis of somKO MEF lines in comparison with wt and mut MEFs. HDAC3 is included as a loading control. Bands of approx. 80kDa and 130kDa are non-specific proteins recognised by the SmcHD1 antibody. Molecular weight markers are shown on the left. **d** Gene de-repression on wt and somKO Xi, presented as % of Xa expression.

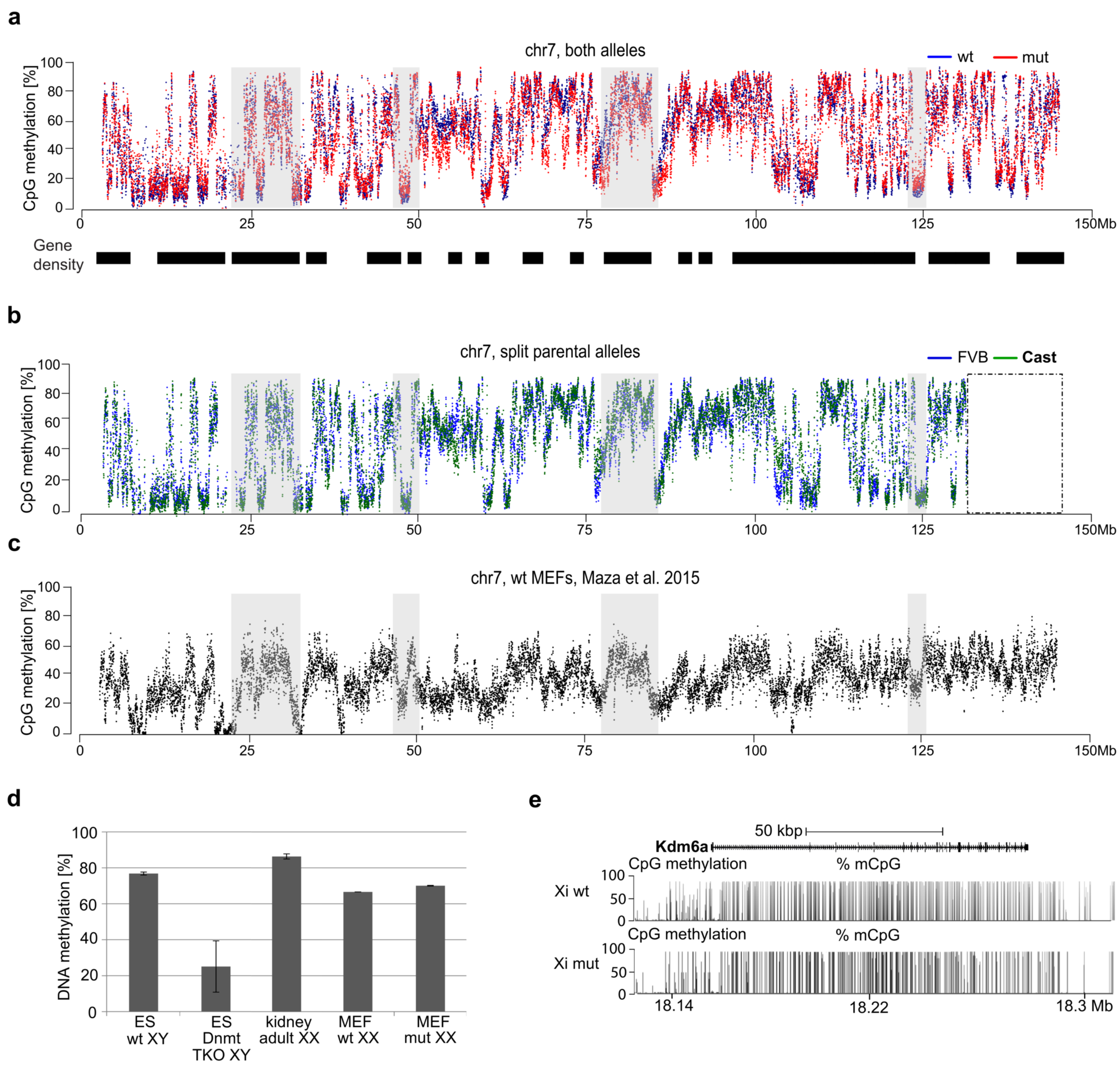

Supplementary Fig. 4 DNA methylation in wt and SmcHD1 mutant MEFs. **a** DNA methylation profile of Chr 7 in wt and SmcHD1 mutant (mut) MEFs plotted as % of mCpG averaged within 10 kb bins. Grey shading depicts example regions of similarity with allele-specific alignment (b) and published MEFs WGBS data set. Bars below the plot indicate regions of high gene density. **b** Allele-specific DNA methylation profiles of wt Chr7 in plotted as % of mCpG averaged within 10 kb bins. Reads aligning to chromosome originating from different strain in blue (FVB) and green (M.m.castaneus). White box depicts region where chromosomes are undistinguishable (possibly due to meiosis). **c** DNA methylation profile of Chr 7 using published MEF WGBS data[38]. **d** Global DNA methylation level of MEF lines used in this study and embryonic stem (ES) cells or adult tissue determined by HPLC. TKO denotes Dnmt1, Dnmt3a, Dnmt3b triple knockout ES cells. **e** Example of DNA methylation profile of known escapee, Kdm6a (UCSC screen shot).

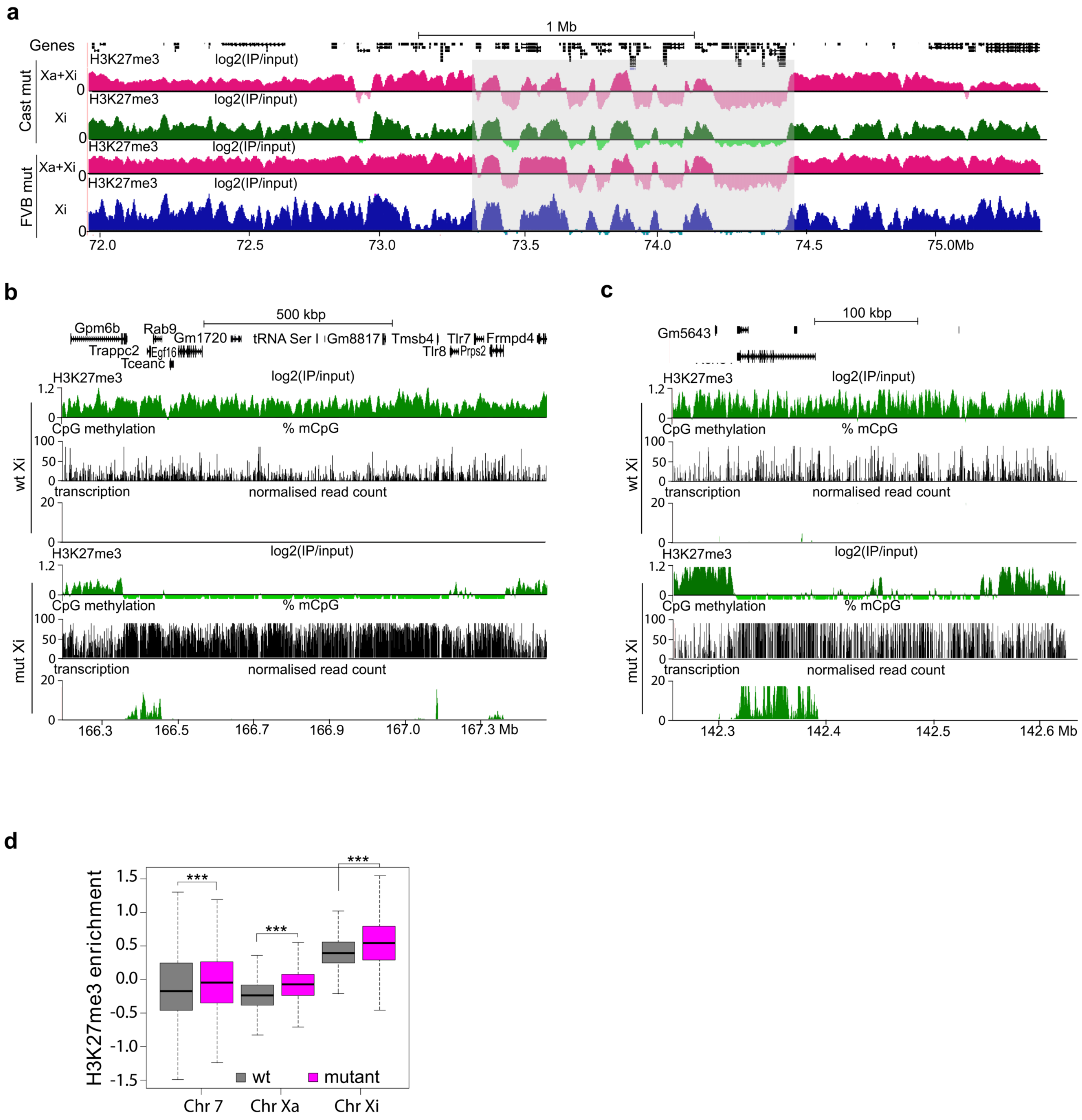

Supplementary Fig. 5 Domains depleted of H3K27me3 in SmcHD1 mutant MEFs. **a** Example of region on chrX with H3K27me3 depletion domains in the mut5 A6 clone used throughout the study with Xi derived from M.m.castaneus (Xa from FVB strain) and in cell line mut5 E1 coming from different parents and with Xi from FVB (Xa from M.m.castaneus). **b** Example showing large domain that is H3K27me3 depleted on the SmcHD1 mutant (mut) Xi and that encompasses both transcribed and silent genes. **c** An example of an H3K27me3 depleted domain in which there are no annotated genes or detectable transcription. **d** H3K27me3 enrichment within 10kb bins on chr7, X (both alleles), Xa and Xi in wild-type and, shown here, germline SmcHD1 mutant MEFs. Boxplots present quartiles, median and outliers. ChIP log2 ratios of IP to input per 500 kb. Significance estimated with Mann-Whitney test (p-value $<10^{-4}$).

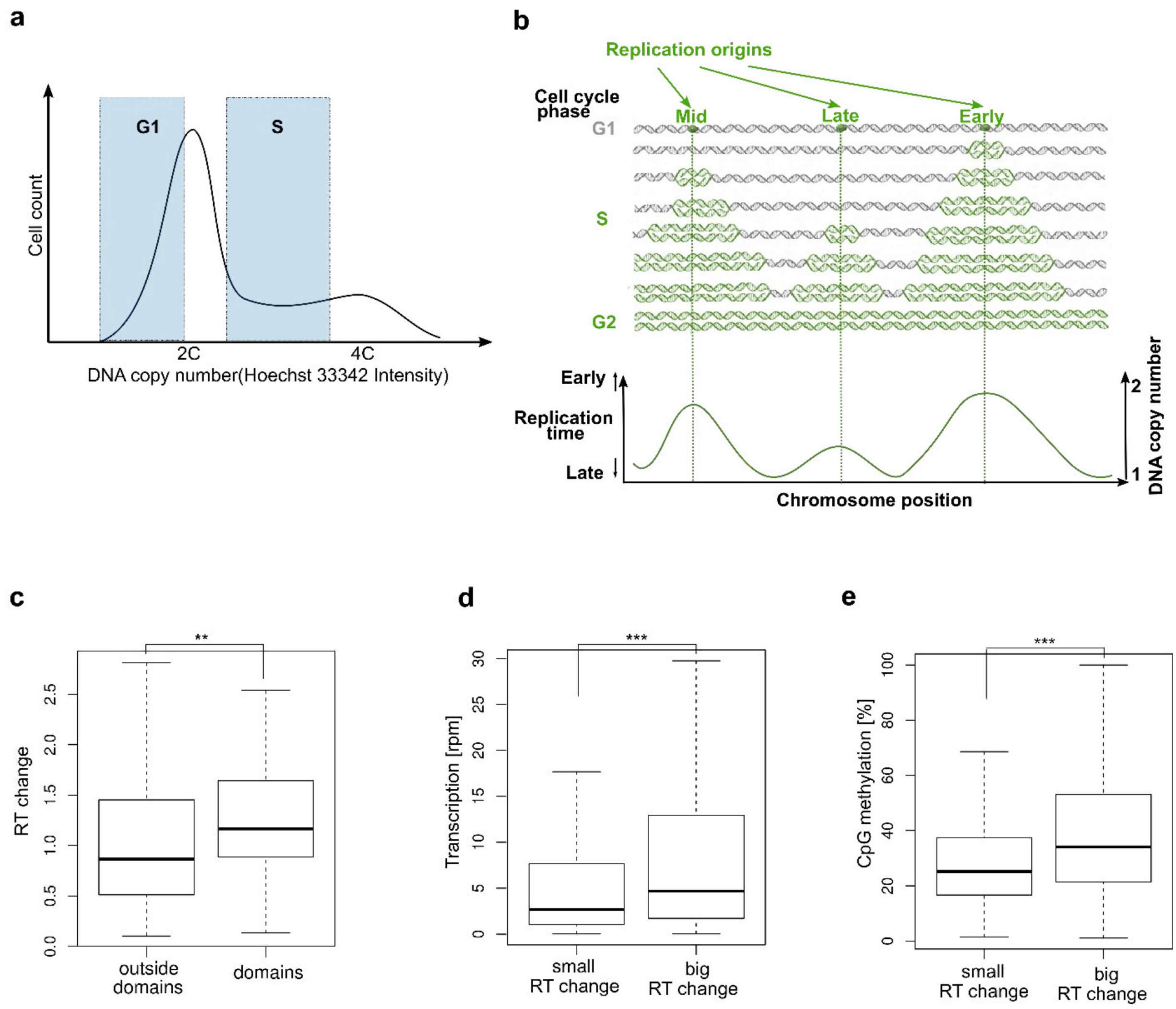

Supplementary Fig. 6 Generation of genome-wide replication timing profiles using Repli-seq. **a** Asynchronously proliferating cells are flow-sorted based on their DNA content (Hoechst 33342) into G1 and S phase fractions. **b** All cells in S-phase have early replicating regions of DNA copied, but regions replicating later are doubled only in cells which are more advanced in genome replication. Therefore in DNA extracts from asynchronous cell populations, early replicating DNA is most abundant. The later replication occurs, the lower is the copy number of the DNA sequence. Thus, S-phase DNA copy number profile obtained with high-throughput sequencing (coverage profiles, PCR-free library prep), normalised with an analogous profile for G1 phase from the same cell population, allows the genome-wide replication timing profile to be obtained. Source: www.amnonkoren.com, with permission of author. **c** RT change: difference in the replication timing shift between Xa and Xi in wt and mut MEFs within H3K27me3 depletion domains and outside of these domains. Replication timing estimated as Z-score of G1/S read densities. Throughout the figure, boxplots present quartiles, median and outliers. Significance estimated with Mann-Whitney test (** $10^{-2}<$ p-value $> 10^{-4}$, *** p-value $< 10^{-4}$). **d** Difference in transcription levels (reads per million from both strands, binned in 10 kb) in region of large (above the median) and small (below the median) RT change. **e** Difference in CpG methylation (% of methylated cytosines binned in 10 kb) in region of large (above the median) and small (below the median) RT change.

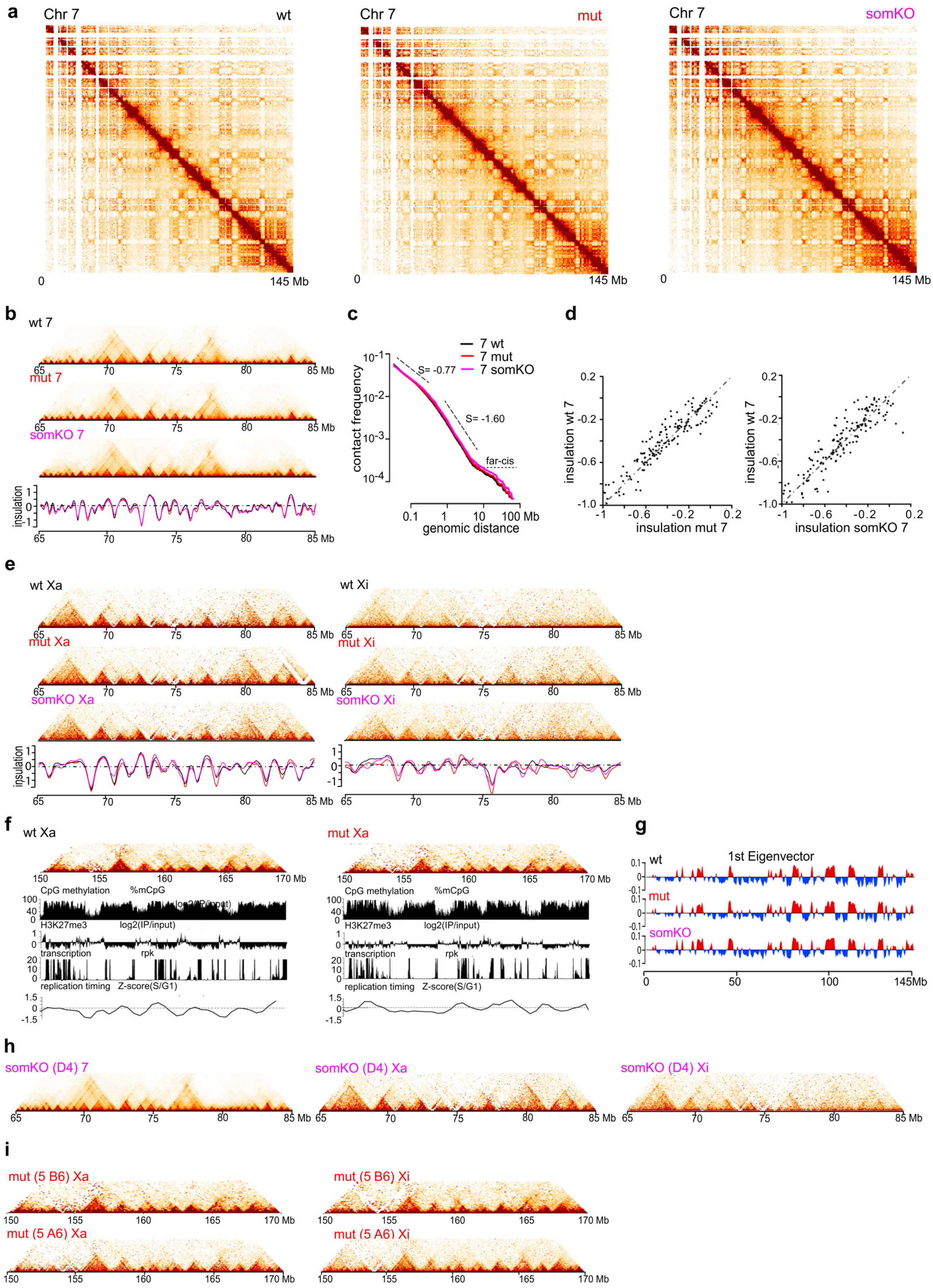

Supplementary Fig. 7 Higher-order structure of autosomes and Chr X in SmcHD1 mutant and wt MEFs. **a** Heatmap depicting Hi-C interactions for Chr 7 of wt, SmcHD1 mutant (mut) and somatic knockout (somKO) MEFs. Interaction matrices were generated from Hi-C

interactions normalised for the available read number per chromosome, KR-balanced and binned into 250 kb bins. **b** Heatmaps presenting local chromatin conformation of two representative 20 Mb regions on Chr 7. Interactions binned into 50 kb bins. **c** Distribution of the distances separating interacting loci for distinct cell lines for chr7. Slopes of linear parts of curves and far-cis interactions indicated as in Fig.5d. **d** Insulation scores at wt chr7 TAD borders calculated for wt vs mut Xi (left) and wt vs somKO MEFs. **e** Local Hi-C interactions within 20 Mb region surrounding "hinge" between the mega-domains on ChrX. Allele-specific Hi-C heatmaps for Xa and Xi of wt and mut MEFs were generated as in Fig. 6D. **f** Similar to Fig.5h, allele-specific Hi-C heatmaps of Xa of the wt and mut MEFs aligned with respective DNA methylation, H3K27me3, transcription and replication timing profiles for the distal 20 Mb of Chr X. **g** First EigenVector values for Xi and Xa of wt, mut and somKO cells. A compartments coloured in red, B compartments in blue. **h** Hi-C heatmap as in Fig.S7b presenting data reproducibility from a different somKO clone (D4) **i** Hi-C heatmap comparing data from independent mut cell lines, mut 5 B6 (100kb bin) and clone mut5 A6 (50 kb bin).

a

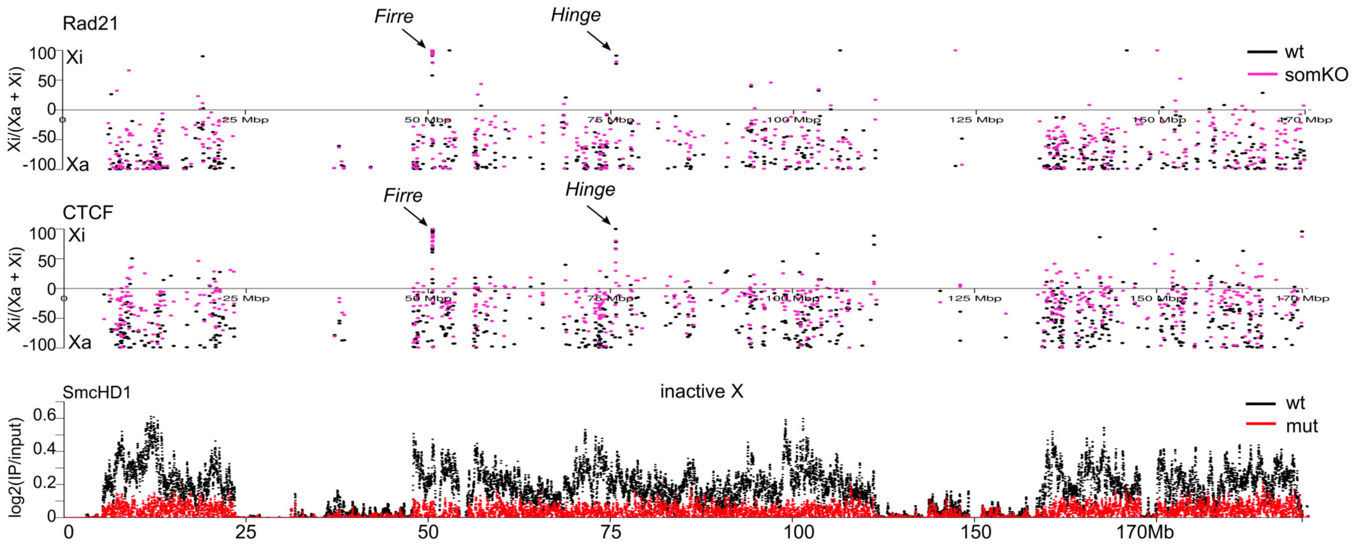

b

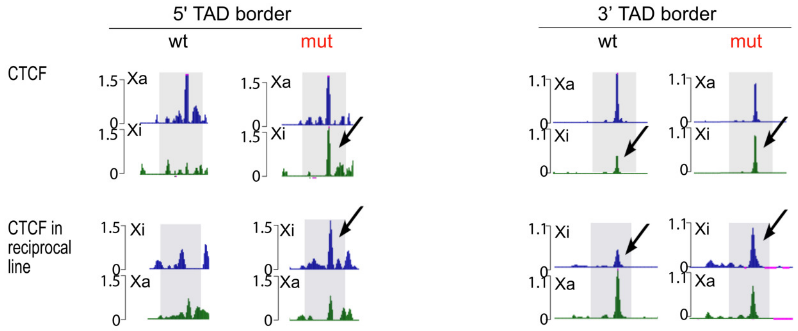

Supplementary Fig. 8 CTCF and Rad21 ChIP-seq analysis. **a** Chromosome-wide occupancy of Rad21 (top panel) and CTCF (middle panel) for Xi or Xa, estimated for distinct peaks occupied in all three cell lines. Values for wt (black) and SmcHD1 somatic knockout (somKO) MEFs (red) calculated as in Fig.6a. Black arrows depict Firre and Hinge loci. Bottom track shows for reference SmcHD1 occupancy profile on the wt Xi as log2(IP/input) in 10 kb bins. **b**. Similarity in allelic skew in the CTCF occupancy in reciprocal wt and SmcHD1 mutant (mut) cell lines. CTCF binding at the TAD borders presented on the Fig. 6.c,d in reciprocal wt and mut mefs in comparison with the original lines used in other experiments. Arrows denote Xi (FVB in reciprocal cell line, M.m.castaneus in the original cell lines used initially). CTCF signal in the 5' border of reciprocal wt cells is not visible due either to low mappability or a SNP mutation.

Name	Application	Sequence 5' → 3'
**SmcHD1_1412**	gRNA	caccgTTACAGTGGACGATCAGCTT
**SmcHD1_1414**	gRNA	caccgCTGCAATCCACAGTATAGAC
**SmcHD1_1415**	gRNA	caccGGCGATCCAGATCAAACATC
**SmcHD1_TNK208**	genotyping/screening KO	GGAAGGTAGAATTTACTAAGTTTTGC
**SmcHD1_TNK227**	Genotyping/screening KO	ATTTAAGGCTTAACTTTAGAGCTG
**SmcHD1_TNK289**	Screening/characterisation KO	TGTTGGAACTAAACTGTTAAACAG
**SmcHD1_TNK290**	Screening/characterisation KO	GGCAGCAGTGTTAGCTGTGG
**SmcHD1_TNK291**	Screening/characterisation KO	AATGAAAGCAGTAGCTTACAGG
**SmcHD1_TNK330**	Screening/characterisation KO	CCAAGGATGGTGTAATATTG
**Uba1XA**	genotyping XX/XY	TGGTCTGGACCCAAACGCTGTCCACA
**Uba1XB**	genotyping XX/XY	GGCAGCAGCCATCACATAATCCAGATG
**Xist_SX20**	genotyping Xist SNP	AGTGGGTGTTCAGGGCGTGG
**Xist_SX21**	genotyping Xist SNP	CTATCCCCTAGTCCTCTGCGG

Supplementary Table 1 Oligonucleotides used in this study

Antibody	Raised in	Source	Reference/ Cat. No.	Dilution for IF	Dilution for WB	Used per IP
**H3K27me3**	Rabbit	Diagenode	C15410069	-	-	5μg
**IgG**	Rabbit	Sigma	M7023	-	-	5μg
**SmcHD1 anti-mouse**	Rabbit	Raised in house	Brideau et al, 2015	1:500	1:1,000	10μl
**Rad21**	Rabbit	Abcam	ab992	-	-	5μg
**CTCF**	Rabbit	Millipore	07-729	-	-	5μl
**H2AK119u1**	Rabbit	Cell Signalling	mAb #8240	1:1,000	-	-
**H3K27me3**	Mouse	Active Motif	61017	1:1,000	-	-
**Alexa 568 anti-mouse IgG**	Goat	Life Technologies	A11031	1:500	-	-
**Alexa 488 anti-rabbit IgG**	Goat	Life Technologies	A11008	1:500	-	-
**Anti-HDAC3 [Y415]**	Rabbit	Abcam	mAb ab32369	-	1:5,000	-
**Anti-rabbit IgG, HPR**	Donkey	Amersham	NA934	-	1:2,000	-
**IRDye 800CW anti-rabbit IgG**	Goat	Li-COR	926-32211	-	1:15,00	

Supplementary Table 2 Antibodies used in this study
